# Low Diversity of Alkhurma Hemorrhagic Fever Virus, Saudi Arabia, 1994–1999

**DOI:** 10.3201/eid1105.041298

**Published:** 2005-05

**Authors:** Rémi N. Charrel, Ali Mohamed Zaki, Mazen Fakeeh, Amany Ibrahim Yousef, Reine de Chesse, Houssam Attoui, Xavier de Lamballerie

**Affiliations:** * Université de la Méditerranée, Marseille, France;; † Dr. Suliman Fakeeh Hospital, Jeddah, Saudi Arabia

**Keywords:** molecular epidemiology, flavivirus, viral hemorrhagic fever

## Abstract

Alkhurma hemorrhagic fever virus (genus *Flavivirus*, AHFV) was recently identified as the agent of a viral hemorrhagic fever in Saudi Arabia and characterized serologically and genetically as a variant genotype of Kyasanur Forest disease virus (KFDV). Since viral diagnosis and vaccine development may be hindered by genetic diversity, this study was intended to address AHFV genetic heterogeneity. Eleven strains isolated from hospitalized patients from 1994 to 1999 in Saudi Arabia were sequenced in the envelope, NS3, and NS5 genes. Homologous sequences were compared and used to look for patterns reflecting specific evolution associated with spatiality, temporality, infection pathway, and disease prognosis. Genetic analyses showed low diversity, which suggests a slow microevolution. Evaluation of divergence times showed that AHFV and KFDV ancestral lineage diverged 66–177 years ago, and the diversity observed within the studied AHFV strains reflected a 4- to 72-year period of evolution.

Alkhurma hemorrhagic fever virus (AHFV) was first isolated in Jeddah, Saudi Arabia, in the 1990s from the blood of a butcher admitted to the hospital with a severe infectious syndrome. To date, 24 cases have been recorded in a 10-year period. Clinical manifestations include fever, headache, retroorbital pain, joint pain, generalized muscle pain, anorexia and vomiting associated with leukopenia, thrombocytopenia, and elevated levels of liver enzymes. In addition, some patients had clinical symptoms of hemorrhagic fever or encephalitis; overall, 5 patients, of 24 infected, died, for a 25% fatality rate (1–4).

AHFV was identified as a flavivirus on the basis of immunofluorescence assay performed with the flavivirus-specific monoclonal antibody 4G2 and polymerase chain reaction (PCR) amplification of a 220-bp genome fragment that exhibited 89% nucleotide (nt) sequence homology with the Kyasanur Forest disease virus (KFDV) NS5 gene. Recently, the complete coding sequence of AHFV was determined; comparative analysis with other tickborne flaviviruses confirmed that AHFV was most closely related to KFDV, and genetic distances suggested that AHFV was a subtype of KFDV (5). In this study, 11 human isolates of AHFV, obtained in a 5-year period, were studied. Partial envelope and NS3 and NS5 genes were sequenced for each isolate and used to conduct detailed genetic analyses. The results of these analyses are presented and discussed.

## Materials and Methods

### Samples

Virus isolation was performed from 1994 to 1999 at the virology laboratory of Dr. Suliman Fakeeh Hospital in Jeddah from blood samples from 11 patients. After centrifugation, serum was injected into suckling mice both intracerebrally or intraperitoneally, and mice were observed twice daily to detect death or signs of illness. Mice showing symptoms were killed, and their brains were harvested, suspended in 10% Hanks balanced salt solution with 20% fetal bovine serum, and centrifuged at 3,000 rpm for 30 min. The supernatant was used to inject tissue culture and for passage in another litter of mice. All isolates included in this study have been passaged twice in mice, except isolate 1176, which was passaged twice in mice, 3 times in Vero cells, once in sheep, and finally once in mice. One hundred microliters of mouse brain suspension was mixed with 900 μL of RNA NOWTM TC-Kit (Biogentex. Inc., Seabrook, TX, USA) and shipped to the Unité des Virus Emergents laboratory in Marseille, France.

### RNA Purification, Amplification, and Sequencing

RNA was purified in a BSL-3 laboratory according to the manufacturer's instructions. Reverse transcription (RT) of virus-specific RNA was carried out at 42°C in a 20-μL reaction that included 11 μL of RNA extract, 200 U of Superscript IITM RNase H-Reverse Transcriptase (Gibco BRL, Life Technologies, Inc., Grand Island, NY, USA), and 1 pmol of primer ALK-NS5R. Truncated noninfectious cDNA molecules were produced. PCR products were generated independently from the envelope, NS3 region, and NS5 region by using ALK-ES/ALK-ER, ALK-NS3S/ALK-NS3R, and ALK-NS5S/ALK-NS5R pairs of primers, respectively. PCR reactions were carried out in a volume of 100 μL that included 10 mmol/L Tris-HCl (pH 9.0), 1.5 mmol/L MgCl_2_, 50 mmol/L KCl, 0.1% Triton X-100, 200 μmol/L each deoxynucleoside triphosphate, 0.2 μmol/L of each primer, 3 μL of cDNA and 1.5 U of Taq DNA polymerase (Promega Corp., Madison, WI, USA). The thermocycler profile was 5 min at 95°C, followed by 35 cycles of 30 s at 95°C, 1 min at 55°C, and 2 min at 72°C, and terminated by a final extension for 7 min at 72°C. PCR products of the expected size were purified from agarose gel slices with the Wizard PCR Preps DNA Purification System (Promega). Both strands of each PCR product were sequenced directly, with the ABI Prism BigDye Terminator Cycle Sequencing Ready Reaction Kit v3.1 on an Applied Biosystems 3730x1 DNA Analyzer (Applied Biosystems, Foster City, CA, USA) at the company Genome Express (Meylan, France).

### Sequence Data and Phylogenetic Analysis

Sequences from the 11 AHFV isolates were compared with homologous sequences representing other tickborne flaviviruses retrieved from the GenBank database: Langat virus (LGTV) (M73835), Powassan virus (POWV) (L06436), deer tick virus (DTV) (AF311056), Omsk hemorrhagic fever virus (OHFV) (AY323489), Kyasanur Forest disease virus (KFDV) (AY323490 for envelope sequence, and personal data for NS3 and NS5 sequence), tick-borne encephalitis virus (TBEV) Neudoerfl strain (U27495), Hypr strain (U39392), Vasilchenko strain (L40361), Sofjin strain (AB062064), and Louping ill virus (LIV) (Y07863). Nonvectored flaviviruses (Rio Bravo virus [NC_003675] and Apoi virus [NC_003676]) were used to root the phylogenetic tree. The nucleotide sequence alignments were generated with Clustal W 1.7 (6). Nucleotide sequence identities were calculated by the pairwise distance algorithm with the MEGA software program (7). Colinearized sequences derived from envelope, NS3, and NS5 regions were used for studying evolutionary mechanisms. Phylogenetic relationships were determined by using the Jukes-Cantor algorithm combined with either neighbor-joining (NJ) or Minimum Evolution methods implemented in MEGA. Maximum parsimony analyses were also performed in MEGA. The robustness of the resulting branching patterns was tested by bootstrap analysis with 500 replications.

### Macroevolution and Microevolution

Previous data estimated the times of divergence between LIV and other member viruses of the tickborne flavivirus complex on the basis of an analysis of the rates of nonsynonymous substitutions within complete E gene sequences (8). A comparable analysis was performed here with the dataset of colinearized partial envelope, NS3, and NS5 sequences obtained as described above. In a first step, it was shown that, within the group of viruses encompassing LIV, TBEVNEU, TBEVSOF, OHFV, LGTV, KFDV, and POWV, rates of nonsynonymous substitutions in colinearized E-NS3-NS5 sequences are a linear function of rates of nonsynonymous substitutions in complete E gene sequences (R^2^ = 0.995). Distances observed by using nonsynonymous sites were calculated according to the Nei and Gojobori algorithm implemented in MEGA (9). This method allowed plotting genetic distances (nonsynonymous substitutions) between colinearized sequences against divergence times calculated by Zanotto et al. (8), which permitted the evaluation of divergence times among AHFV isolates and between AHFV and KFDV. Using the same method, we estimated divergence times between LIV and other tickborne flaviviruses selected in this study.

## Results

### Epidemiologic Findings

**Figure 1:**
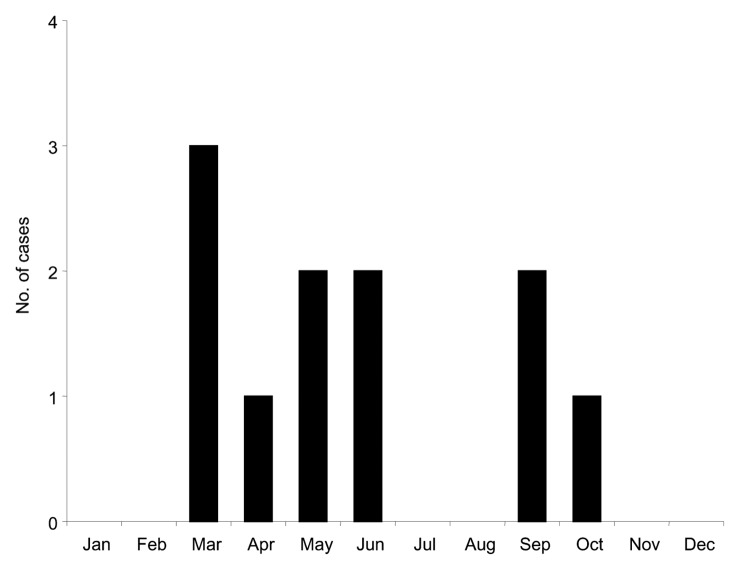
Annual distribution of the 11 cases of Alkhurma hemorrhagic fever virus infections in Saudi Arabia, 1994–1999.

Available epidemiologic data are presented in Table 1. The 11 isolates were recovered during a 5-year period; the first case was recorded in May 1994 and the last in June 1999. Analysis of the seasonal distribution of cases showed that they occurred in 2 peaks, lasting from March to June and from September to October, respectively (Figure 1). All cases occurred in patients originating from Mecca or Jeddah, 2 cities in Saudi Arabia 75 km apart, representing a zone of 5,000 km^2^.

**Table 1:** Epidemiologic data available for the 11 male patients infected with Alkhurma hemorrhagic fever virus (AHFV)

AHFV isolate	Date of isolation	Nationality	Occupation	Source of infection	Origin	Outcome
87	May 1994	Egyptian	Butcher	Wound	Mecca	Recovery*
228	May 1994	Egyptian	Butcher	Wound	Mecca	Death
1176	September 1995	Egyptian	Butcher	Wound	Mecca	Death
MOS	September 1995	Egyptian	Butcher	Wound	Jeddah	Recovery*
1209	October 1995	Saudi	Soldier	Camel raw milk	Jeddah	Recovery*
5975	June 1997	Saudi	Driver	Camel raw milk	Jeddah	Death
7344	March 1998	Saudi	Engineer	Tick bite	Jeddah	Recovery*
7466	March 1998	Egyptian	Butcher	Wound	Jeddah	Recovery*
7471	March 1998	Egyptian	Butcher	Wound	Jeddah	Recovery*
7586	April 1998	Saudi	Student	Tick bite	Jeddah	Death
9518	June 1999	Eritrean	Poultry worker	Camel raw milk	Jeddah	Recovery*

*Recovery without sequelae.

### Sequence Analysis

RT-PCR amplification using primers ALK-ES/ALK-ER, ALK-NS3S/ALK-NS3R, and ALK-NS5S/ALK-NS5R produced 742-bp, 757-bp, and 723-bp products, respectively (Table 2). Once primer sequence was excluded, the respective lengths of the sequences included in the study were 699 nt, 713 nt, and 685 nt. These sequences were deposited in GenBank under accession numbers AY727543–AY727575. All AHFV sequences were of the same length, and an optimal alignment was obtained without incorporating any gap. The genetic diversity observed among the 11 strains of AHFV included in this study was up to 0.4%, 0.6%, and 0.9% in E, NS3, and NS5 regions, respectively, and p distance of nonsynonymous substitutions per nonsynonymous sites reached 0% in the envelope, 0.19% in the NS3 gene, and 0.77% in NS5. A total of 21 nt positions were variable, 6 of them associated with a modification of the encoded amino acid (Table 3). Fourteen of 28 observed mutations were nonsynonymous and therefore affected the protein sequence. Thirteen were located in the NS5 at nucleotide positions NS5-1679, NS5-1693, NS5-1720, NS5-1930, and NS5-2096, and 1 was located in NS3 at nucleotide position NS3-1177. The multipassaged 1176 strain did not develop more mutations than the low-passage isolates. We sequenced the homologous regions of KFDV strain P9605 (corresponding to the first human isolate, isolated from blood in 1957 by Dandavate at the Virus Research Center at Vellore field station) for comparative analysis (R.N. Charrel and X. de Lamballerie, unpub. data). In the 3 regions, the genetic heterogeneity observed between the sequences of the 11 AHFV isolates and KFDV strain was 7.3%–7.6%, 6.6%–7.2%, and 8.2%–8.8% at the nucleotide level for E, NS3, and NS5 regions, respectively.

**Table 2:** Positions of primers used for PCR amplification and sequencing of AHFV genome and resulting sequences*

Primer name	Sequence	Position, per AHFV prototype sequence	PCR product size (bp)	Sequence used for analysis†
ALK-ES	GGATATGTGTATGATGTCAATAA	1342–1364	742	699
ALK-ER	GCTGCAGTTCAACGAAACCT	2083–2064		
ALK-NS3S	CAATGAAGCTTATGTTAGTAGC	5064–5085	757	713
ALK-NS3R	CACAAAATCTGGCTTCTCTTCT	5820–5799		
ALK-NS5S	AGCAAATCCTGCGTTATCTGA	9308–9328	723	685
ALK-NS5R	GTCCCTGCGGGACCCAG	10030–10014		

*AHFV, Alkhurma hemorrhagic fever virus; PCR, polymerase chain reaction. †Primers excluded.

**Table 3:** Mutated positions in the sequences of the 11 AHFV isolates included in the study*

Position/gene†	Coding sequence	Strain										
		228	87	mos	1176	1209	5975	7471	7466	7344	7586	9518
E												
480	1323	A	A	A	A	A	A	A	G	A	A	A
513	1356	G	G	G	G	G	G	G	A	G	G	G
579	1422	T	T	T	T	T	T	T	T	T	T	C
1062	1905	A	A	A	A	A	G	A	A	A	A	A
NS3												
600	1323	C	C	C	C	C	C	C	T	C	C	C
687	1356	T	T	T	T	T	T	T	T	C	C	T
714	1422	G	G	G	G	G	G	G	G	A	G	G
600	1905	T	T	T	T	T	C	T	T	T	T	T
687	1323	C	C	C	C	C	C	T	C	C	T	C
714	1356	A	A	A	A	G	A	A	A	A	A	A
600	1422	T	T	T	T	T	C	T	T	T	T	T
687	1905	A	A	A	A	A	A	**G**	A	A	A	A
NS5												
1079	1323	G	G	G	G	**C**	G	G	G	G	G	G
687	1356	C	C	C	C	C	C	C	C	C	**T**	C
714	1422	T	T	**C**	T	T	T	T	T	T	T	T
600	1905	C	C	C	C	C	C	C	C	T	C	C
687	1323	A	A	A	A	A	A	A	A	A	A	G
714	1356	G	G	G	G	**A**	**A**	**A**	G	G	**A**	**A**
600	1422	T	T	T	T	T	T	T	T	T	T	C
2096	1905	A	A	A	A	**T**	**T**	**T**	A	A	**T**	**T**
2244	9783	C	C	C	C	T	C	C	C	C	C	C

*AHFV, Alkhurma hemorrhagic fever virus. †By reference to the nucleotide sequence of AHFV prototype strain deposited in GenBank under accession number AF331718; shaded cell indicates mutated position; underlined nucleotides indicate nonsynonymous mutations.

### Phylogenetic Analysis

**Figure 2:**
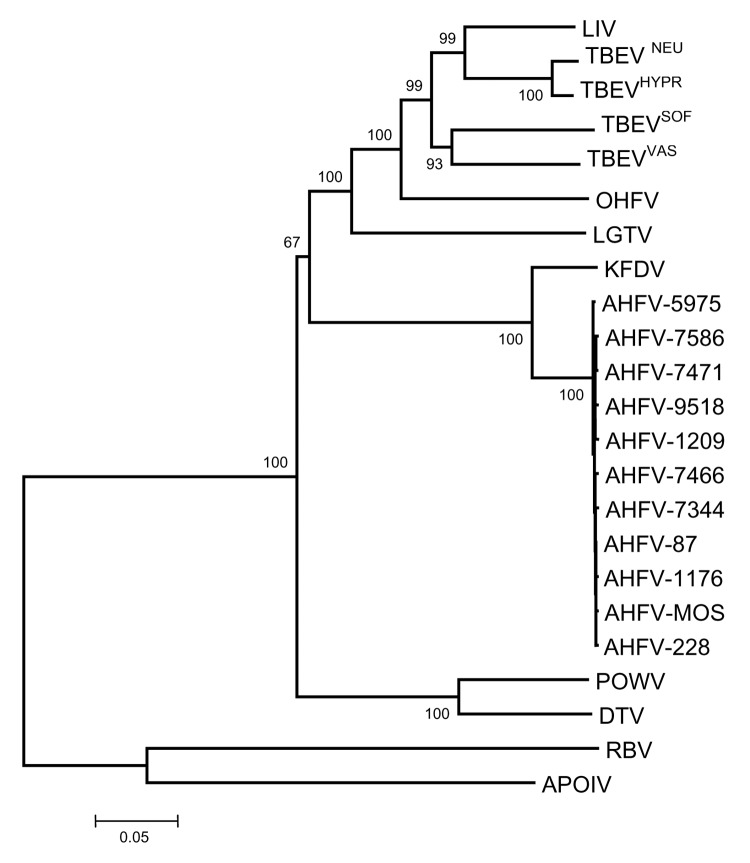
Phylogenetic analysis of Alkhurma hemorrhagic fever virus isolates and selected tickborne and nonvectored flaviviruses based on a 2097-nucleotide (nt) sequence constituted by the colinearization of E, NS3, and NS5 sequences (699, 713, and 685 nt, respectively). Distances and groupings were determined by the Jukes-Cantor algorithm and neighbor-joining method with the MEGA 2.1 software program (7). Bootstrap values are indicated and correspond to 500 replications.

Phylogenetic analyses performed independently with envelope, NS3, and NS5 gene sequences did not provide an accurate picture of the recently published topology deduced from complete coding sequences (5,10). Accordingly, a dataset of colinearized sequences was used to increase the discrimination of analysis and provide branching patterns concordant with complete sequence-based analysis. Such procedures were recently reported to reflect adequately complete genome analysis of West Nile virus strains (11). Figure 2 represents the phylogenetic reconstruction based on the analysis of E-NS3-NS5 colinearized sequences. All phylogeny algorithms used provided very similar results in term of branching patterns. All AHFV isolates clustered together. Their closest relative was KFDV. The existence of a more divergent lineage common to AHFV and KFDV was supported by a 100% bootstrap value.

## Discussion

Little information exists on seasonal patterns and host preferences of the tick species circulating in Saudi Arabia. However, extensive investigations of the most closely related virus, KFDV, have established that 2 species of ticks (*Ixodes petauristae*, *I. ceylonensis*) are involved in viral transmission and that they exhibit different seasonal peaks; similarly, each developmental stage has a peak of activity that corresponds to different seasons. Whether AHFV has similar features is unknown, but if so this finding could account for 2 peaks of cases during the year (8 cases from March to June, 3 cases from September to October). Two additional cases reported through ProMED in 2002 and 2004 (2,4) also occurred in March and April, thus reinforcing the evidence of a peak in frequency in the spring. Camels and sheep are believed to be the hosts that replicate AHFV, but other mammals may be involved in the natural cycle. Recent reports posted on the ProMED Web site suggest that since 1999, additional cases have occurred (3). Accordingly, AHFV is maintained through a cycle that needs to be clarified through veterinary and entomologic surveillance programs. As discussed, analysis of genetic data provided no evidence for multiple introductions of the virus in Saudi Arabia during the period studied; however, this must be confirmed with sequence data covering a larger time period.

Although the number of reported cases is low, case histories suggest that AHFV may have infected humans through various routes (oral, direct contact, tick bite), as previously demonstrated for other flaviviruses vectored by ticks (12). Based on a questionnaire administered by the physician when a patient was admitted, 3 different modes of infection were considered probable, i.e., skin wound (n = 6), tick bite (n = 2), and consumption of unpasteurized raw camel milk (n = 3). Working as a butcher in a slaughterhouse was the occupation with the most exposure; the 6 butchers most probably acquired AHFV infection through skin abrasions or wounds and contact with sheep-infected blood. Although, the 5 other patients did not have clear occupational exposures, 2 of them (or their relatives) reported tick bites shortly before the episode, and the 3 others (or their relatives) reported raw milk consumption. Therefore, these 5 patients may have acquired AHFV infection through tick bite or raw milk consumption, as previously documented for tick-borne encephalitis virus (12,13).

Although the human cases were acquired from different sources and through different routes, the clinical and biological features were similar; whether asymptomatic infection may occur is not known and would require seroepidemiologic studies. From 1999 to 2004, the ProMED Web site reported on 3 occasions a total of 9 cases of infection; at least 2 were fatal. The overall rate of death observed with AHFV (6/24, 25%) appears to be much higher than that currently reported for KFDV (3.0%–8.9%) (14,15) and stable during the period 1994–2004.

The genetic variability between the different isolates was low regardless of 1) time of the year during which the strain was recovered, 2) disease symptoms in the infected patients, 3) presumed route of infection, and 4) environment in which the patient lived. This pattern was observed for KFDV isolates in India, although these conclusions were established from antigenic methods rather than sequence analyses (14). This evolutionary pattern is consistent with the existence of a recent common ancestral lineage for all AHFV isolates characterized in this study. This lineage went through a period of in situ evolution in this region. The fact that all strains were recovered from humans, after suckling mouse inoculation, could bias the results by acting as a genetic filter. Whether AHFV genetic diversity is as low as implied in this study remains to be seen. This point will only be resolved when strains obtained from cattle, camels, and ticks from Saudi Arabia and neighboring countries are studied. However, because these strains were transmitted to humans through distinct pathways, they do not likely represent a specific genetic cluster associated with a specific pathogenicity.

**Figure 3:**
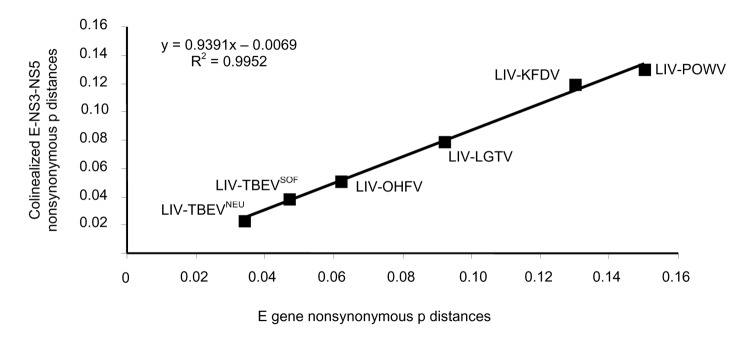
Correlation between p distances at nonsynonymous sites by using the Nei and Gojobori method (9) obtained from complete E gene and colinearized E-NS3-NS5 sequences. As previously reported (8), all distances were calculated between Louping ill virus (LIV) and tick-borne encephalitis virus Neudoerfl strain, tick-borne encephalitis virus Sofjin strain, Omsk hemorrhagic fever virus (OHFV), Langat virus (LGTV), Kyasanur Forest disease virus (KFDV), and Powassan virus (POWV), respectively.

Evolution of AHFV and closely related tickborne flaviviruses was addressed on the basis of previous estimation of the times of divergence of flavivirus species (8) (Figure 3). By using the same method, we estimated that KFDV and AHFV diverged 66–177 years ago. By comparison, the divergence between POWV and DTV, according to the same algorithm, was estimated to have occurred 275–393 years ago, and divergence between TBENEU and LIV occurred 291–411 years ago (estimated 364–498 years ago [8]). Accordingly, the divergence between KFDV and AHFV appears to be a recent event in the evolution of tickborne flaviviruses, comparable with the divergence observed between isolates of LIV (8). Regarding the common ancestor to the AHFV isolates included in this study, and when one takes the life cycle of ticks into account, the algorithm indicated that strains diverged 4–72 years ago, which implies a limited number of generations between this ancestor and the strains currently circulating in Saudi Arabia.

The high genetic similarity between all AHFV strains makes designing and developing specific and sensitive RT-PCR diagnostic assays possible. Close antigenic properties are also important for vaccine development. Among the many issues that merit future investigations, cross-protection conferred by KFDV and commercially available tick-borne encephalitis virus vaccines should be tested since slaughterhouse workers appear to rank high on the risk scale and therefore may be the first population to benefit from this information.

Seroepidemiologic studies are needed in various population groups to determine the extent of AHFV infection and its geographic distribution in the Middle East peninsula (Yemen, Oman). Studies are needed to achieve a better understanding of the natural history of this virus but its pathogenicity in humans, specifically the prevalence of asymptomatic and symptomatic cases. The most important issues to resolve are the origin of the virus, how it is dispersed, and how it came to be in Saudi Arabia so that disease control strategies can be devised.
